# Haematology and serum biochemistry in captive Australian native murids: black-footed tree-rat (*Mesembriomys gouldii*) and greater stick-nest rat (*Leporillus conditor*)

**DOI:** 10.1186/s40064-016-3111-7

**Published:** 2016-09-02

**Authors:** Melissa L. Tulk, Hayley J. Stannard, Julie M. Old

**Affiliations:** 1 School of Science and Health, Hawkesbury, Western Sydney University, Locked Bag 1797, Penrith, NSW 2751 Australia; 2 School of Life and Environmental Sciences, Charles Perkins Centre, University of Sydney, Sydney, NSW 2006 Australia

**Keywords:** Australian native rodent, Blood, Health, Leukocyte morphology

## Abstract

The black-footed tree-rat (*Mesembriomys gouldii*) and greater stick-nest rat (*Leporillus conditor*) are near threatened and vulnerable native Australian murids. There is a paucity of health and welfare knowledge for these species and native murids in general. In this paper we aimed to address this deficiency in knowledge by describing some key haematological and blood biochemistry parameters for these species. Haematology and blood biochemistry data were obtained from clinical histories of the two murid species held in captivity at Taronga Zoological Park, Mosman, Australia. The data were analysed to establish confidence intervals for each parameter available and leukocyte morphology described. White blood cell counts were higher in females than males. Both species also had high neutrophil:lymphocyte ratios (tree-rat ratios were almost even). Haematocrit was higher in male stick-nest rats than females. Differential leukocyte counts and leukocyte morphology was consistent with previous descriptions in other murids and between individuals. Blood biochemistry values were unremarkable except for the high level of globulin in stick-nest rats. The values provided in the study will add to the knowledge of health data for murids in captivity and aid captive and natural management of Australian native murids.

## Background

The continuous decline of Australian mammals has occurred since European settlement (Burbidge and McKenzie 1989) and is influenced by a combination of factors. The Muridae family, the only family of rodents found in Australia, is not exempt from this decline. They currently account for up to 40 % of all Australian mammalian species (Breed and Ford 2007), with at least 57 currently recognised (Van Dyck and Strahan 2008). Animals with a larger body mass are able to maintain a sufficient population density, decreasing the risk of extinction, leaving the smaller animals, such as murids (in the critical weight range of 25–500 g) (Johnson and Isaac 2009) with a higher rate of extinction risk (Burbidge and McKenzie 1989).

The near threatened (IUCN 2014) black-footed tree-rat (*Mesembriomys gouldii*) is one of Australia’s largest rodents weighing 550–800 g (Breed and Ford 2007). The arboreal species is only found in three remote locations: Kimberly/mainland Northern Territory, north Queensland and Melville Island. Due to their solitary behaviour and large home ranges (>60 ha) (Breed and Ford 2007; Griffiths et al. 2002) population numbers are difficult to determine but are estimated at 10,000–12,000 in each location. Residing in tree hollows, this species is vulnerable to fires, habitat loss, fragmentation and degradation. Predation is also a major threat and has contributed to the decline of this species with a 30–50 % population decline in the last decade (Woinarski et al. 2014).

The greater stick-nest rat (*Leporillus conditor*) is a vulnerable species (IUCN 2014) previously found across much of the semi-arid and arid zone of Australia (Van Dyck and Strahan 2008). Currently, there is only one natural population located on Franklin Island in the Nuyts Archipelago, South Australia (Robinson 1975). The species has been bred in captivity and successfully re-introduced to a conservation reserve near Roxby Downs (South Australia), Reevesby Island (South Australia) (Arid Recovery 2007), St Peters Island (South Australia) and Salutation Island (Western Australia) (Morris 2000). Weighing 180–450 g, the ground dwelling stick-nest rat is vulnerable to predation by native and introduced predators.

Due to constant threats to the long-term population survival of the two species, in situ conservation efforts alone are not sufficient to ensure their survival. Long-term survival of these wild populations relies on maintaining captive populations, and maintaining the health of these animals is critical in the captive setting. It is also very often assumed that the health parameters of native murids are the same as domesticated laboratory murids despite the two groups of murids being distinctly separated taxonomically at the sub-Family level (Van Dyck and Strahan 2008). In addition, the causes and consequences of disease in native murid species are generally poorly understood, particularly for Australian species, and long-term data sets are required for health and disease studies. For example, there is generally a paucity of information regarding the haematology, serum biochemistry and leukocyte morphology for most Australian native murids, with only a few having previously been documented for captive specimens (Bradley et al. 1988; Kemper et al. 1987; Monamy 1995; Old et al. 2005, 2007). Haematology and serum biochemistry values for the black-footed tree-rat and greater stick-nest rat are needed as differences in physiology and life history traits compared to other murids, means values for other species are not directly comparable.

This study aimed to establish baseline confidence intervals for haematology, serum biochemistry and leukocyte morphology for these two species living in captivity. The information obtained may aid the conservation of black-footed tree-rats and greater stick-nest rats, and increase our knowledge of the biology of captive Australian native murids.

## Methods

### Study animals

The animals used in this study were all from the captive colonies housed at Taronga Zoological Park, Mosman, NSW, Australia. These animals were housed in species-specific enclosures. Male tree-rats were held in a 19.7 m^2^ enclosure, and the females in a 3.1 m^2^ enclosure. All stick-nest rats were held in a 17.5 m^2^ enclosure. The enclosures contained dirt/leaf litter or sand substrate with structural complexities provided by flora and browse. The animals were fed on a mixed diet of insects, vegetation, seeds, nuts, and fruit.

All samples were obtained from the park’s medical reports from October 1995 to February 2015. Animals were categorized as ‘healthy’ or ‘unhealthy’ based on the clinical notes provided. Blood samples were taken as part of routine health checks, quarantine procedures or enquiries based on clinical symptoms of illness.

### Blood collection and analysis

The animals were removed from their enclosure and taken to the park’s in-house wildlife hospital for blood collection and analysis. Animals were anesthetized with isoflurane/O_2_ during blood collection though an induction chamber and maintained through a facemask. Extraction location of the blood samples varied between species and individuals from the saphenous, femoral and jugular vein/artery.

White blood cell (WBC) counts (×10^9^ cells/L) were determined using improved Neubaur counting chambers by Taronga pathology at time of blood collection, if not, WBC counts were manually determined at Western Sydney University using the original blood smears. The leukocyte, platelet, differential leukocyte count and leukocyte morphology were determined from Diff Quik (Thermo Fisher, Scoresby, Victoria) stained blood smears. Digital images of the leukocytes were obtained using a BX60 microscope (Olympus, Japan) with a ProgRes C14 camera (Jenoptik, Germany).

Once extracted, blood biochemistry values were measured using a Reflotron Instrument and IDEXX Vet Test (IDEXX Laboratories, Rydalmere, NSW), until late November 2010, when the REM systems VetScan2 and associated consumables (REM Systems, North Ryde, NSW) were used. Rotor plates were used to determine the values of 26 different blood parameters. The parameters included haematological parameters: estimated platelets (per/HOIF), haemoglobin (HGB) (g/L) and haematocrit (HCT) (%). In addition we analysed the serum biochemical parameters: Gamma-glutamyl transpeptidase (GGT) (U/L), lipase (U/L), chloride (mmol/L), total carbon dioxide (CO_2_) (mmol/L), creatine kinase (U/L), glucose (mmol/L), blood urea nitrogen (BUN) (mmol/L), creatinine (mmol/L), calcium (mmol/L), phosphorus (mmol/L), sodium (mmol/L), potassium (mmol/L), total protein (g/L), albumin (g/L), globulin (g/L), alanine transaminase (ALT) (U/L), total bilirubin (μmol/L), amylase (U/L), alkaline phosphatase (ALP) (U/L) and aspartate aminotransferase (AST) (U/L).

### Data analysis

All values from ‘healthy’ individuals were analysed, including those samples obtained from the same individual, at different times. We conducted the analysis in this way because of the paucity of information available on Australian native murids generally. By utilising all samples available it provides a more accurate representation of the true values for each parameter, and hence provides more accurate confidence intervals, than if we only included one value randomly chosen for each individual. Using the Analysis Toolpak available on Microsoft Excel 2010, 90 % confidence intervals for each parameter were determined. Due to the large number of different conditions and diseases affecting the murids’ classified as clinically ‘unhealthy’, none of the parameters for these individuals were statistically analysed. We have however included the parameters of those individuals classified as clinically ‘unhealthy’ in this paper, to provide an indication of those values that are more likely to suggest poor health.

## Results

In this study, data from 37 captive individuals (some sampled multiple times) were included, from samples collected over two decades. The past two decades saw a large change in how blood was analysed and what animal healthcare professionals investigated when analysing blood. A number of parameters were only tested on one individual and were therefore excluded from the results, as it was not regarded as representative of the species. Each individual sample was classified as ‘healthy’ or ‘unhealthy’, as determined by the accompanying clinical notes. Statistical comparisons were only made on ‘healthy’ animals. Animals that were ‘unhealthy’ varied in their conditions and were therefore not collectively analysed, and were included as individual samples rather than as part of a population sample.

A total of 32 black-footed tree-rat samples consisting of 25 ‘healthy’ (18 female and 7 male) and seven ‘unhealthy’ (3 female and 4 male) samples, aged between 0.6 and 7.1 years old were used in this study. Greater stick-nest rat samples were obtained from 25 ‘healthy animals’ (18 female and 7 male), aged between 0.3 and 6.3 years old and one ‘unhealthy’ female aged 2.8 years (Table 1).

**Table 1:** Haematology values for healthy black-footed tree-rats

Parameter	Mean ± SD	90 % CI lower limit	90 % CI upper limit	Male mean ± SD	Female mean ± SD
WBC count (×10^9^ cells/L)	18.70 ± 13.87 (n = 22)	13.84	23.57	8.76 ± 4.68 (n = 7)	23.34 ± 14.37 (n = 15)
Haemoglobin (g/L)	143.05 ± 22.88 (n = 21)	135.19	150.90	145.57 ± 19.79 (n = 7)	141.79 ± 23.46 (n = 15)
Haematocrit	43.77 ± 5.78 (n = 22)	41.75	45.80	44.86 ± 6.91 (n = 7)	43.27 ± 5.36 (n = 14)
Neutrophil (%)	44.11 ± 19.92 (n = 22)	37.13	51.09	45.70 ± 21.10 (n = 7)	43.37 ± 20.06 (n = 15)
Lymphocyte (%)	48.54 ± 20.71 (n = 23)	41.43	55.64	43.43 ± 18.69 (n = 7)	50.77 ± 21.76 (n = 16)
Monocyte (%)	4.08 ± 7.78 (n = 21)	3.08	5.08	2.99 ± 1.55 (n = 6)	4.52 ± 3.07 (n = 15)
Eosinophil (%)	6.63 ± 5.55 (n = 19)	4.53	8.72	9.67 ± 7.13 (n = 6)	5.22 ± 4.28 (n = 13)
N:L ratio	1.39 ± 1.34 (n = 22)	1.86	2.96	1.74 ± 2.09 (n = 7)	1.22 ± 0.92 (n = 15)
Est. platelets (/HOIF)	28.08 ± 24.68 (n = 16)	17.93	38.23	15.67 ± 10.89 (n = 6)	35.53 ± 28.02 (n = 10)

*N:L* neutrophil to lymphocyte ratio, *CI* confidence intervals

### Haematology

Results for haematology of ‘healthy’ black-footed tree-rats and greater stick-nest rats are shown in Tables 2 and 3. Mean total WBCs were higher in female tree-rats compared to males. The majority of female tree-rats between the ages of 2.5–5.0 years had total WBCs counts over 18 × 10^9^ cells/L (up to 52.4 × 10^9^ cells/L); with younger females (<2.5 years) below 10.0 × 10^9^ cells/L and all male tree-rats below 20.0 × 10^9^ cells/L. Stick-nest rat mean total WBCs were higher in females compared to males. HCT mean levels were higher in male stick-nest rats compared to females. Tree-rats had similar mean percentages of neutrophils (44.1 %) and lymphocytes (48.5 %), while the stick-nest rats had a higher mean percentage of neutrophils (63.9 %) compared to lymphocytes (32.0 %). The range of N:L ratios in both species was large; tree-rats 0.1–6.1 ($\bar{x}$ = 1.4) and stick-nest rats 0.3–11.5 ($\bar{x}$ = 2.2).

**Table 2:** Haematology values for healthy greater stick-nest rats

Parameter	Mean ± SD	90 % CI lower limit	90 % CI upper limit	Male mean ± SD	Female mean ± SD
WBC count (×10^9^ cells/L)	11.25 ± 4.87 (n = 25)	9.65	12.85	9.00 ± 4.28 (n = 11)	13.01 ± 4.56 (n = 14)
Haemoglobin (g/L)	111.78 ± 21.95 (n = 9)	99.74	123.81	103.33 ± 21.87 (n = 6)	128.67 ± 7.85 (n = 3)
Haematocrit^a^	34.08 ± 5.07 (n = 25)	32.00	36.00	36.73 ± 3.79 (n = 11)	32 ± 4.97 (n = 14)
Neutrophil (%)	63.89 ± 17.36 (n = 25)	58.18	69.60	57.01 ± 16.17 (n = 11)	69.29 ± 16.31 (n = 14)
Lymphocyte (%)	32 ± 16.22 (n = 25)	26.66	37.33	37.29 ± 15.30 (n = 11)	27.83 ± 15.69 (n = 14)
Monocyte (%)	4.49 ± 3.79 (n = 18)	3.02	5.96	5.66 ± 4.52 (n = 9)	3.33 ± 2.36 (n = 9)
Eosinophil (%)	2.08 ± 1.52 (n = 13)	1.39	2.78	1.84 ± 1.49 (n = 6)	2.30 ± 1.52 (n = 7)
N:L ratio	3.23 ± 3.00 (n = 25)	27.89	46.22	1.85 ± 0.86 (n = 11)	4.32 ± 3.58 (n = 14)
Est. platelets (/HOIF)	37.05 ± 24.29 (n = 19)	2.00	4.00	28.67 ± 10.48 (n = 6)	44.60 ± 30.03 (n = 10)

*N:L* neutrophil to lymphocyte ratio, *CI* confidence interval

**Table 3:** Blood biochemistry values for healthy black-footed tree-rats

Parameter^a^	Mean ± SD	90 % CI lower limit	90 % CI upper limit	Male mean ± SD	Female mean ± SD
GGT (U/L)	12.55 ± 3.46 (n = 2)	8.52	16.58	–	12.55 ± 3.46 (n = 2)
Chloride (mmol/L)	106.00 ± 1.41 (n = 2)	104.36	107.64	–	106.00 ± 1.41 (n = 2)
Total CO_2_ (mmol/L)	20.35 ± 10.96 (n = 2)	7.60	33.10	–	20.35 ± 10.96 (n = 2)
Cholesterol (mmol/L)	0.95 ± 0.21 (n = 2)	0.70	1.19	–	0.95 ± 0.21 (n = 2)
Glucose (mmol/L)	12.06 ± 7.32 (n = 20)	9.37	14.75	12.45 ± 6.77 (n = 5)	11.93 ± 7.71 (n = 15)
BUN (mmol/L)	6.62 ± 2.24 (n = 21)	5.82	7.42	7.03 ± 1.18 (n = 6)	6.45 ± 2.56 (n = 15)
Creatinine (mmol/L)	46.28 ± 16.37 (n = 17)	39.77	52.79	50.00 ± 12.35 (n = 4)	45.14 ± 17.63 (n = 13)
Calcium (mmol/L)	2.56 ± 0.18 (n = 9)	2.47	2.66	2.55 ± 0.20 (n = 3)	2.57 ± 0.18 (n = 6)
Phosphate (mmol/L)	1.55 ± 0.45 (n = 11)	1.32	1.77	1.69 ± 0.42 (n = 4)	1.46 ± 0.47 (n = 7)
Sodium (mmol/L)	142.00 ± 3.77 (n = 9)	139.93	144.07	140.67 ± 2.31 (n = 3)	142.67 ± 4.37 (n = 6)
Potassium (mmol/L)	4.06 ± 1.41 (n = 11)	3.36	4.76	3.25 ± 2.21 (n = 4)	4.52 ± 0.44 (n = 7)
Total protein (g/L)	56.33 ± 13.48 (n = 12)	49.93	62.73	51.20 ± 19.04 (n = 5)	60.00 ± 7.33 (n = 7)
Albumin (g/L)	46.73 ± 11.76 (n = 11)	40.90	52.56	50.50 ± 7.94 (n = 4)	44.57 ± 13.56 (n = 7)
Globulin (g/L)	11.73 ± 11.48 (n = 11)	6.03	17.42	5.00 ± 6.22 (n = 4)	15.57 ± 12.37 (n = 7)
ALT (U/L)	60.03 ± 34.61 (n = 14)	44.81	75.24	61.48 ± 9.49 (n = 5)	59.22 ± 43.58 (n = 9)
AST (U/L)	95.04 ± 58.06 (n = 10)	63.20	126.88	76.70 ± 29 (n = 3)	102.90 ± 71.41 (n = 7)
Total bilirubin (μmol/L)	4.61 ± 2.07 (n = 10)	3.53	5.68	6.33 ± 2.31 (n = 3)	3.87 ± 1.58 (n = 7)
Amylase (U/L)	1996.89 ± 2215.15 (n = 9)	780.71	3213.06	1037 ± 39.40 (n = 3)	2476.83 ± 2653.75 (n = 6)
ALP (U/L)	82.69 ± 72.95 (n = 13)	49.41	115.97	54 ± 36.30 (n = 4)	95.44 ± 83.03 (n = 9)

*CI* confidence interval

^a^Lipase (U/L) and Creatine kinase (U/L) were not included due to numerical irregularities

### Morphology of blood cells

**Fig. 1:**
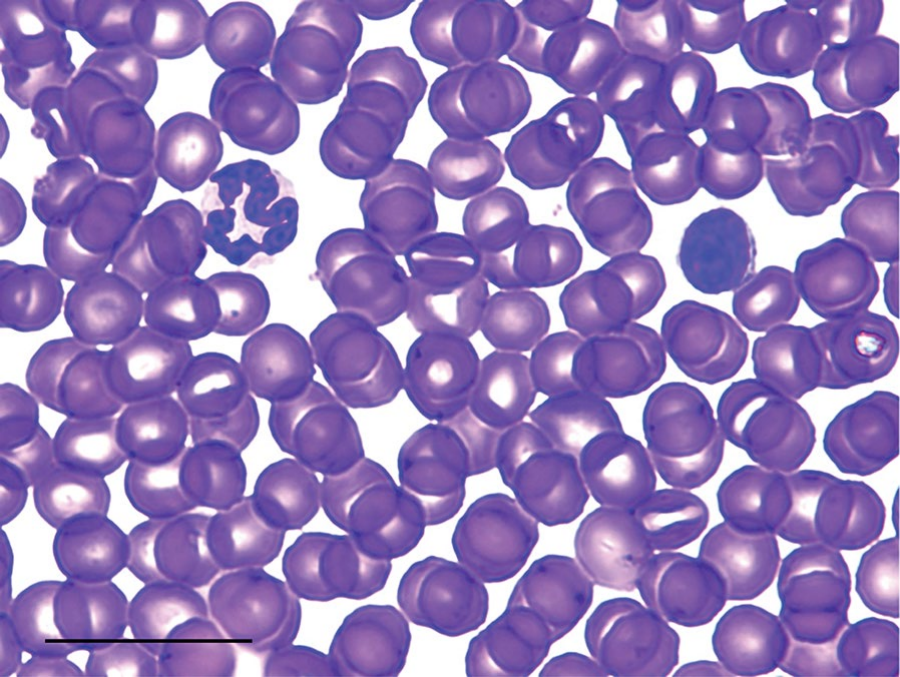
A male black-footed tree-rat multi-lobed neutrophil (*left*) with two distinct lobes and a lymphocyte (*right*). *Scale bar* 20 μm

**Fig. 2:**
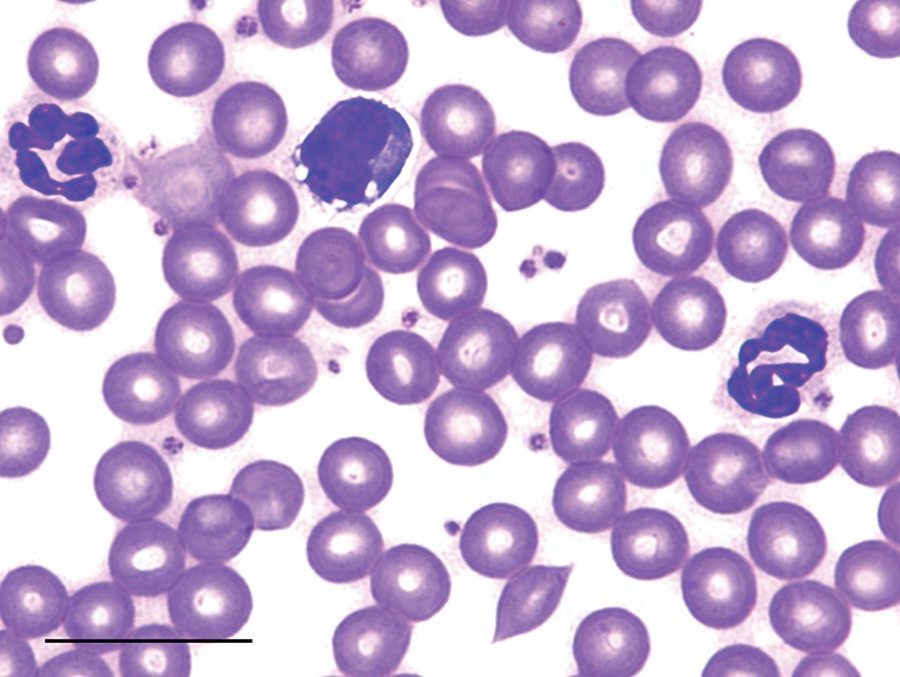
Two neutrophils (*left* and *right*) and a vacuolated monocyte (*center*) from a male black-footed tree-rat. *Scale bar* 20 μm

Only neutrophils, lymphocytes, and monocytes were observed in the blood smears of both species. No eosinophils or basophils were observed in blood smears. The nucleus of the neutrophils of the tree-rat were hypersegmented and polymorphonuclear, containing between 3 and 4 large lobes, and ranging from 5 to 10 μm (Figs. 1, 2). Tree-rat lymphocytes measured 5–12 μm (Fig. 2). Tree-rat monocytes measured 5–11 μm in diameter and had reticular chromatin and a moderate level of basophilic cytoplasm with vacuoles (Fig. 2).

**Fig. 3:**
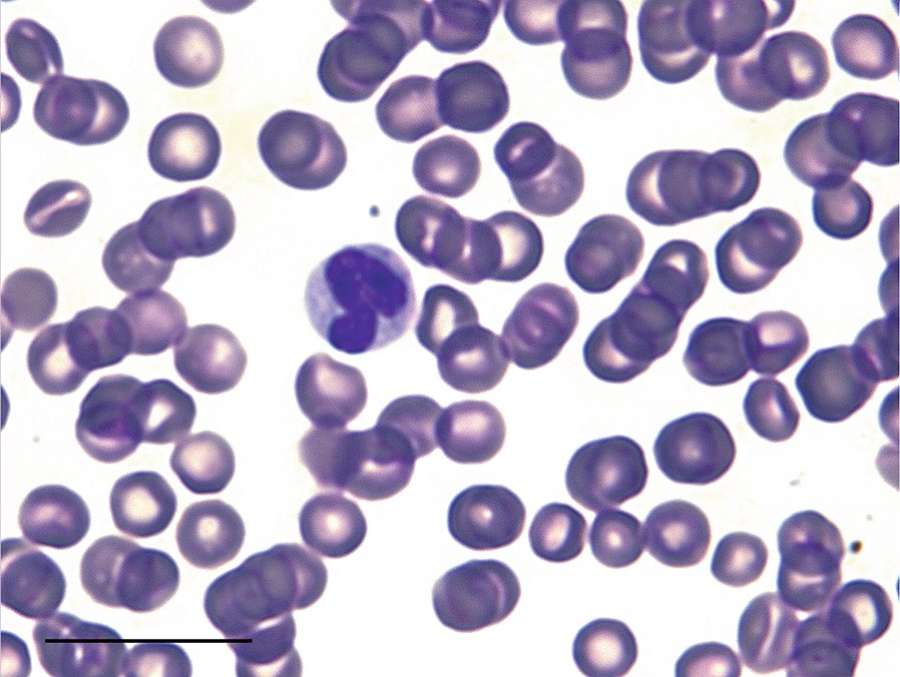
Multi-lobed neutrophil from a male greater stick-nest rat. *Scale bar* 20 μm

**Fig. 4:**
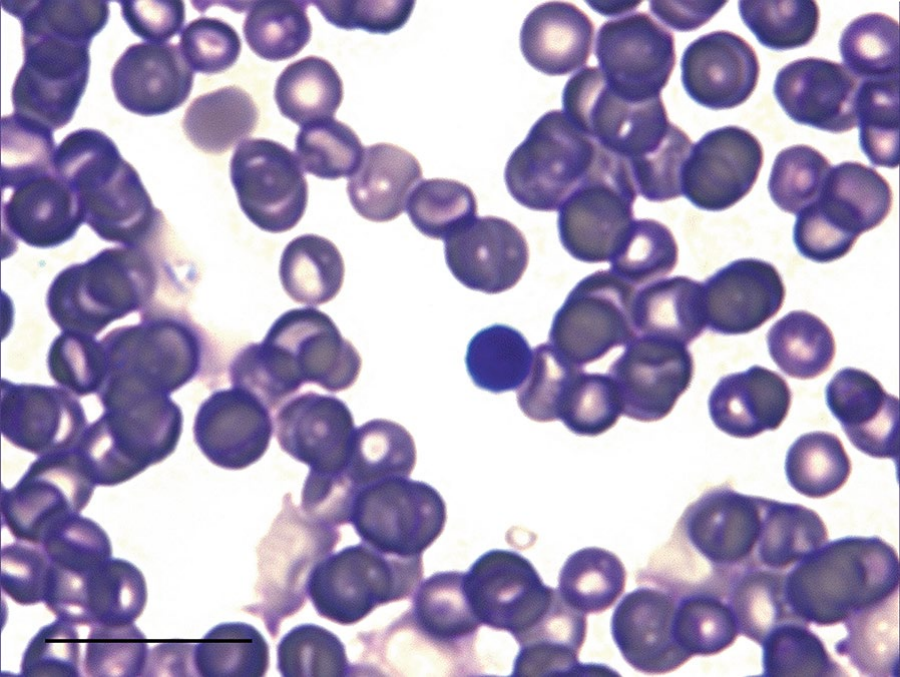
A male greater stick-nest rat lymphocyte with very little cytoplasm visible. *Scale bar* 20 μm

**Fig. 5:**
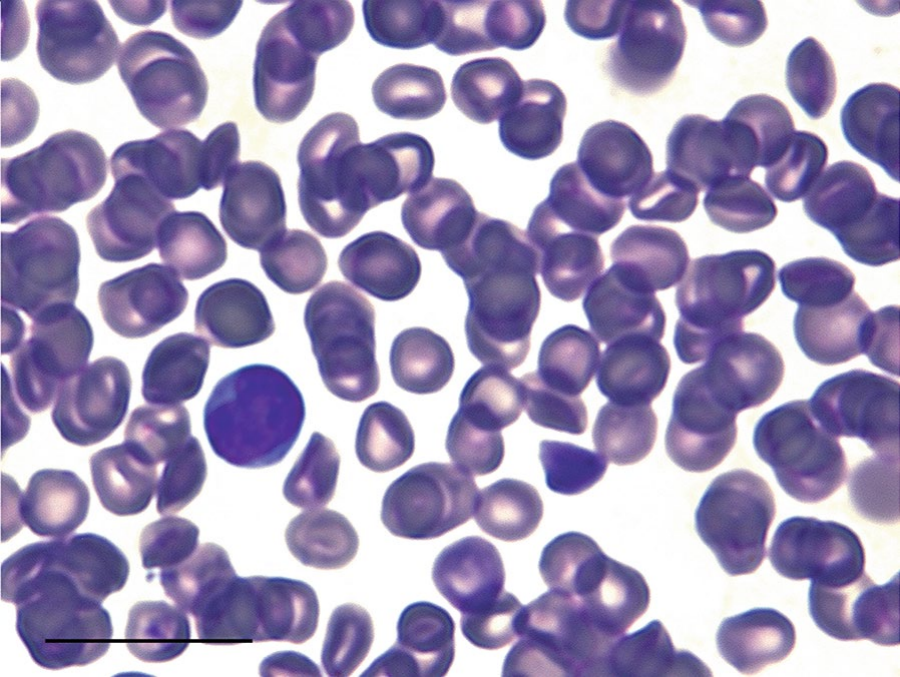
Monocyte from a male greater stick-nest rat with slightly indented nucleus to form a horseshoe shape. *Scale bar* 20 μm

The greater stick-nest rat neutrophils observed had a darkly stained nucleus containing minimal definitive segmentation, with 3–4 large lobes, and varied in size between 9 and 21 μm in diameter (Fig. 3). Occasional neutrophils with an annular nucleus were seen. Lymphocytes were 6–10 μm in diameter (Fig. 4). The monocytes measured 6–17 μm in diameter and the nucleus was indented giving it a horseshoe appearance (Fig. 5).

### Serum biochemistry

Serum biochemistry values for the black-footed tree-rat (Table 4) and greater stick-nest rat (Table 5) were similar to previously reported murids for most parameters analysed. Stick-nest rats however had a high globulin concentration (Table 5) in comparison to other murids (Bradley et al. 1988; Kemper et al. 1987; Monamy 1995; Old et al. 2005, 2007; Thrall et al. 2012). All other values are reported in Table 5.

**Table 4:** Blood biochemistry values for healthy greater stick-nest rats

Parameter	Mean ± SD	90 % CI lower limit	90 % CI upper limit	Male mean ± SD	Female mean ± SD
Creatinine kinase (U/L)	123.00 ± 55.93 (n = 14)	92.60	322.54	123.00 ± 55.93 (n = 6)	271.00 ± 328.58 (n = 8)
Glucose (mmol/L)	6.36 ± 3.15 (n = 19)	5.63	9.47	6.36 ± 3.15 (n = 9)	8.62 ± 6.15 (n = 10)
BUN (mmol/L)	14.77 ± 3.02 (n = 21)	12.00	16.00	14.77 ± 3.02 (n = 9)	13.47 ± 6.96 (n = 12)
Creatinine (mmol/L)	46.09 ± 17.72 (n = 20)	39.57	52.60	44.45 ± 8.57 (n = 8)	47.18 ± 21.71 (n = 12)
Phosphate (mmol/L)	2.14 ± 0.73 (n = 10)	1.91	2.88	2.14 ± 0.73 (n = 4)	2.57 ± 1.02 (n = 6)
Potassium (mmol/L)	4.32 ± – (n = 4)	3.56	4.13	4.32 ± – (n = 1)	3.69 ± 0.24 (n = 3)
Total protein (g/L)	59.20 ± 8.23 (n = 11)	51.62	61.29	59.20 ± 8.23 (n = 5)	54.17 ± 10.30 (n = 6)
Albumin (g/L)	23.00 ± 5.69 (n = 11)	16.20	23.80	23.00 ± 5.69 (n = 5)	17.5 ± 8.18 (n = 6)
Globulin (g/L)	30.20 ± 2.23 (n = 11)	29.56	37.34	30.20 ± 2.23 (n = 5)	36.17 ± 9.62 (n = 6)
ALT (U/L)	55.74 ± 21.32 (n = 17)	46.53	64.39	55.74 ± 21.32 (n = 8)	55.21 ± 23.29 (n = 9)
AST (U/L)	52.87 ± 29.44 (n = 15)	51.12	74.75	52.87 ± 29.44 (n = 7)	71.74 ± 22.96 (n = 8)
Total bilirubin (μmol/L)	8.55 ± – (n = 3)	–	–	8.55 ± – (n = 1)	8.55 ± – (n = 2)
ALP (U/L)	208.69 ± 140.21 (n = 13)	144.73	272.66	227.50 ± 97.43 (n = 6)	192.57 ± 166.77 (n = 7)

*CI* confidence interval

**Table 5:** Haematology values of unhealthy murids

Species	Tree-rat I	Tree-rat II	Tree-rat III	Tree-rat IV	Tree-rat V	Tree-rat VI	Tree-rat VII	Stick-nest rat I
Gender	Female	Female	Male	Male	Male	Male	Male	Female
Age (years)	2.91	1.85	3.28	2.66	2.57	2.63	2.05	2.76
WBC count (×10^9^ cells/L)	5.39	6.93		5.94	10.56	7.26	9.70	13.31
Haemoglobin (g/L)	150	128		158	148			101
Haematocrit	45	42	46	46	50	31	36	33
Neutrophil (%)	50	22.94	56.00	37.04	53.03	80.03	62.99	79.04
Lymphocyte (%)	33	72.01	33.00	43.94	35.04	14.05	20	16
Monocyte (%)	10	4.04	11.00	10.94	3.03	4.96	12.99	3.01
Eosinophil (%)	7	1.01		8.08	9	0.10	4.02	2.03
N:L ratio	1.52	0.32	1.70	0.84	1.51	5.70	3.15	4.94
Est. platelets (/HOIF)		10		35	30	35	15	35
Illness	Anorexia, Cataracts	Hyphema, Cataracts	Cataracts	Weight loss	Weight loss	Hypopyon	Hypopyon	Dyspnea

*N:L* neutrophil to lymphocyte ratio

### Unhealthy animals

Individual murids were classified as ‘healthy’ or ‘unhealthy’ based on the notes provided in their medical reports. Although no statistical comparisons were possible, we were able to identify some observational differences between animals classified as ‘healthy’ and ‘unhealthy’. For example, one ‘unhealthy’ tree-rat with hypopyon noted in the medical report, had elevated glucose, creatinine, globulin and ALT, and low BUN and albumin concentrations compared to ‘healthy’ animals (Table 6). Another ‘unhealthy’ male tree-rat said to have lost weight had elevated levels of phosphate and potassium, and lower levels of globulin and ALP (Table 6). Both of these male tree-rats had a lower total WBC and lymphocyte count and elevated neutrophils compared to the ‘healthy’ tree-rats (Table 2). The animal with hypopyon also had an elevated N:L ratio. The ‘unhealthy’ stick-nest rat had lower levels of glucose, BUN, creatinine, phosphate, ALT, and ALP than that of the ‘healthy’ stick-nest rats. This stick-nest rat also had elevated platelets and high N:L ratio (Table 6).

**Table 6:** Blood biochemistry values of unhealthy murids

Species	Tree-rat I	Tree-rat III	Tree-rat IV	Tree-rat V	Tree-rat VII	Tree-rat VIII	Stick-nest rat I
Gender	Female	Male	Male	Male	Male	Female	Female
Age (years)	2.91	3.28	2.66	2.57	2.05	2.89	2.76
Lipase (U/L)					20.00		
Chloride (mmol/L)					5.51		
Creatinine kinase (U/L)					134.00		
Glucose (mmol/L)	9.30	7.20	9.04	13.44	24.19	28.80	4.54
BUN (mmol/L)	6.30	6.40	7.00	8.00	6.39	6.10	8.28
Creatinine (mmol/L)	57.00	51.00	57.02	55.96	73.99	39.00	32.97
Calcium (mmol/L)	2.77	2.76	9.84	2.35		2.91	2.63
Phosphate (mmol/L)	1.58	0.68	1.58	2.11	2.60	3.13	1.69
Sodium (mmol/L)	146.00	141.00	145.00	136.00		147.00	149.00
Potassium (mmol/L)	4.40	4.80	3.80	5.60		4.70	4.00
Total Protein R (g/L)	62.00	54.00		47.00			47.00
Total Protein (g/L)	66.00	57.00	52.00	52.00	64.00	67.00	54.00
Albumin (g/L)	42.00	53.00	52.00	41.00	24.00	51.00	24.00
Globulin (g/L)	24.00	3.00		11.00	42.00	16.00	30.00
ALT (U/L)	83.00	83.00	66.00	63.00	188.00	199.00	20.00
AST (U/L)					218.00		
Total bilirubin (μmol/L)	5.00	6.00	6.00	4.99.00		5.00	4.99.00
Amylase (U/L)	1121.00	931.00	959.00	804.00		919.00	390.00
ALP (U/L)	60.00	62.00	66.00	22.00	104.00	80.00	99.00
Triglycerides (mmol/L)					35.00		
Illness	Anorexia, Cataracts, Hyphema	Cataracts	Weight loss	Weight loss	Hypopyon	Anorexia, Cataracts, Hyphema	Dyspena

## Discussion

In comparison to other captive murids, the captive Australian native tree-rats and stick-nest rats presented differences in their leukocyte morphology, haematology and serum biochemistry. The haematology and serum biochemistry values were relatively consistent between individuals, despite the use of different analysis equipment and regardless of some differences in collection methods between individuals. WBC counts were higher in females in both species. Both species also had high N:L ratios (tree-rat ratios were almost even). HCT was higher in male stick-nest rats than females. Differential leukocyte counts and leukocyte morphology was consistent with previous descriptions in other murids and between individuals. Blood biochemistry values were unremarkable except for the high level of globulin in stick-nest rats when compared to previous murid research (Bradley et al. 1988; Kemper et al. 1987; Monamy 1995; Old et al. 2005, 2007; Thrall et al. 2012).

Healthy specimens of both species had elevated total WBC counts in comparison to the other murids (Bradley et al. 1988; Kemper et al. 1987; Monamy 1995; Old et al. 2005, 2007; Thrall et al. 2012). Tree-rats had a mean WBC count that was almost double that reported previously for murids, while the stick-nest rats were within the expected range for murids, but at the higher end. Stick-nest rats had a higher WBC count when compared to other murids (Bradley et al. 1988; Kemper et al. 1987; Monamy 1995; Old et al. 2005, 2007; Thrall et al. 2012) and had a small standard deviation, suggesting the values are likely to be a true indication of ‘healthy’ stick-nest rat WBC counts. The differences in tree-rat mean WBC counts were different between the two sexes, females having higher counts. A larger sample size is needed to accurately determine species reference values (Table 2).

Both species had neutrophilia, as animals were classified as ‘healthy’ and did not show signs of inflammation, the cause of the condition can be assumed to be physiologic as a result of epinephrine or from stress (Harvey 2012). Neutrophils, usually make up 20–30 % (Provencher Bolliger and Everds 2012) of leukocytes. In tree-rats (44 %) and stick-nest rats (64 %) numbers of neutrophils were much higher than anticipated. Lymphocytes are usually the predominant leukocyte and can be as high as 70–80 % of the differential WBC count in the laboratory mouse (Provencher Bolliger and Everds 2012), however in the black-footed tree-rat lymphocytes were just below 50 % and made up 32 % of all WBCs in the greater stick-nest rats.

High neutrophil to lymphocyte ratios are useful indicators of poor health or stress (Old et al. 2005). On average both species had high N:L ratios, possibly a result of neutrophilia. A fifth of the stick-nest rats were skewed (6.6–11.5), while all other ratios were <4.0, which may account for the high mean ratio. Compared to other captive murids (Bradley et al. 1988; Kemper et al. 1987; Monamy 1995; Old et al. 2005, 2007; Thrall et al. 2012), both species had very high N:L ratios, with tree-rats three times and stick-nest rats six times larger than previously reported murid N:L values.

Both species were rarely handled or removed from their enclosure for any medical procedure. The stress of being handled prior to anaesthesia may explain the irregularities in the values as it can increase the number of neutrophils (Hedrich 2012). Anaesthesia, specifically isoflurane, can have an effect on the percentage of neutrophils found in C3H mice, with 30 min exposures leading to a 15.4 % reduction in the number of circulating WBCs, and specifically a 26.9 % reduction in neutrophils up to 48 h after exposure (Colucci et al. 2013; Jacobsen et al. 2004). Exposure to 4 % isoflurane, if administrated for a duration longer than 5 min may also have had an effect on erythrocytes parameters (Nahas and Provost 2002). The length of time the murids in this study were under anaesthesia is unknown.

The morphological appearance of leukocytes in the two species was similar to that described previously for other murids including the brown rat (*Rattus norvegicus*) (Thrall et al. 2012), plains rat (*Pseudomys australis*), spinifex hopping-mice (*Notomys alexis*) (Old et al. 2005) and the central rock-rat (*Zyzomys pedunculatus*) (Old et al. 2005). Neutrophils of both species in this study were larger in diameter when compared to the house mouse (*Mus musculus*) and brown rat (Thrall et al. 2012). Lymphocyte size greatly fluctuates from the size of erythrocytes to neutrophils (Thrall et al. 2012). Both species’ lymphocytes did not exceed the size of neutrophils. Monocyte size and morphology were similar to that previously described for other murid species (Bradley et al. 1988; Kemper et al. 1987; Monamy 1995; Old et al. 2005, 2007).

The low numbers of eosinophils and basophils was not unexpected. Eosinophil numbers are normally only elevated under certain conditions such as eosinophilia during an allergic response or in individuals with parasites (Harvey 2012). As the two species in this study were both from captive populations it is unlikely they would have had high parasite loads (due to regular treatment), and if allergic reactions were evident, would likely have been recorded in the clinical notes. In mammals, basophils are generally never found in high numbers and in some species can be absent (Latimer 2011).

Globulin values include levels of enzymes, antibodies, and fibrous and contractile proteins. The stick-nest rat had a mean of 30.2 g/L globulin, 8.9–17.8 g/L above the current reported murid range (Bradley et al. 1988; Kemper et al. 1987; Monamy 1995; Old et al. 2005, 2007; Thrall et al. 2012). The cause or effect of high globulin in rodents has not been investigated in detail. However in humans, high globulin can indicate chronic inflammation, an infectious disease, leukaemia, diseases of the liver or kidneys, or an autoimmune disease (Willard and Tvendten 2012). Stick-nest rats over the age of 4 years did display a higher globulin level than their younger counterparts, presumably as they had been exposed to more pathogens than the younger animals. As the expected longevity of free-ranging stick-nest rats is 4 years (Jackson 2007), advanced age (or the wide range of ages of murids in this study) is a reasonable explanation for these high values.

ALP is associated with measurements of skeletal growth and can be used as an indicator of age, with levels decreasing as the animal reaches adulthood (Calabuig et al. 2010). Tree-rat ALP was higher in older animals than younger animals and was not consistent with previous murid values (Bradley et al. 1988; Kemper et al. 1987; Monamy 1995; Old et al. 2005, 2007; Thrall et al. 2012). Stick-nest rat ALP values were low in young individuals, peaked around 2.5 years, and dropped again when animals reached 4 years. In quolls (Stannard et al. 2013) and other murid species (Old et al. 2005) ALP levels varied greatly between individuals. High ALP has been seen as an effect of captivity in the black vulture (*Aegypius monachus*), as well as poor health (Villegas et al. 2002). Whilst higher ALP values have also been reported in healthy captive southern hairy-nosed wombats (*Lasiorhinus latifrons*) compared to wild wombats (Gaughwin and Judson 1980). A larger number of samples with a wider range of ages are needed to determine the reasoning behind the variability in the results and whether captive management is affecting the ALP values of these species.

## Conclusions

Comparative fundamental descriptions of the morphology, relative numbers of leukocytes, and the serum biochemistry of two native Australian murids were provided in this paper to establish a baseline for presumably healthy individuals living in captivity. Our data indicated the values had some variation when comparing genders; however further data is required to determine how age influences blood parameters in these species, specifically in WBC counts in both species as well as N:L ratios/percentages and HCT in stick-nest rats. Compared to other captive murids stick-nest rats had higher levels of globulin and requires further investigation. Nevertheless, the confidence intervals established provide a basis for monitoring the health status of captive individual black-footed tree-rats and greater stick-nest rats and aid the long-term survival of these captive murid populations. The cause, consequence and impact of disease in native murid species remain poorly understood, and further long-term data sets are required to fully understand health and disease in these species, as well as blood samples from wild individuals.
